# Long‐term monitoring of common spadefoot toad activity in a European steppe using barn owl pellets

**DOI:** 10.1186/s40709-021-00133-w

**Published:** 2021-02-12

**Authors:** Lukas Landler, Katharina Stefke

**Affiliations:** 1 Institute of Zoology, University of Natural Resources and Life Sciences, Gregor-Mendel-Straße 33/I, Vienna, 1180 Austria; 2 Natural History Museum Vienna, Mammal Collection, Burgring 7, Wien, 1010 Austria

**Keywords:** *Pelobates fuscus*, Seewinkel, Neusiedler See, Amphibian ecology, Yearly activity

## Abstract

**Background:**

One third of the worldwide amphibian species are threatened, therefore, efficient monitoring efforts are needed. Amphibians which adopt a hidden lifestyle, such as the common spadefoot toad, are often missed with standard surveying efforts. Spadefoot toads can be identified in regurgitated pellets of the barn owl, which provides an effective way to estimate toad activity. In our study we analyzed frequency of spadefoot toad remains from 2004 to 2016 in a steppe landscape in eastern Austria.

**Methods:**

We used an automated model selection procedure together with a GLM analysis using a zero inflated error Poisson distribution, to analyze the presence of *Pelobates fuscus* in barn owl pellets. All analyses were done in the statistical software R, and the scripts to reproduce our results are available within this publication. Our approach may provide a template for other researchers to use for their own pellet data.

**Conclusions:**

Our analysis suggested that activity of the common spadefoot toad is mainly influenced by rainfalls, while time of the year and temperature had small but significant effects. Interestingly, our data confirmed the possibility of a second breeding period in summer, triggered by heavy rainfalls. There were no indications for a population decrease in the observed years and locations. Our study shows that barn owl pellets can be used effectivley to assess pelobatid activity in an area. This might constitute a useful monitoring tool for conservation management for amphibians.

## Background

Worldwide amphibians are among the most threatened vertebrate groups and an estimated 1/3 of them are threatened [1, 2]. In many cases, habitat destruction and degradation, land-use change but also the chytrid fungus are major threats [3–5]. Following the global trend also in Austria, at least half of the native amphibian species are threatened [6]. However, data on the general ecology and seasonal activity of amphibian species is needed, to assess extinction risks and population status. Conservation management efforts need to be able to distinguish between inactivity or extinction of a certain taxa at specific locations. For some amphibians, data on ecology and activity are easy to obtain, because such amphibians typically stay in, or close to, their breeding areas most of the year. In contrast, many amphibians live a more discrete life, e.g. staying buried in the soil for most of the year and only being active during favorable conditions. One such example is the common spadefoot toad (*Pelobates fuscus*), a widespread, however, in Austria critically endangered, pelobatid toad [6]. This toad is threatened by the intensification of agriculture in their remaining habitats, which leads to breeding pond loss and increased use of pesticides [7]. Despite its wide distribution range in Europe, ecological studies are rare. Due to its hidden lifestyle, it can easily remain undetected using standard monitoring procedures [6]. Spadefoot toads are typically night-active and remain buried in the soil during unfavorable conditions.

There has been some discussion concerning the taxonomic status of the spadefoot toads in Europe [8–10]. It has been argued that there is evidence of several cryptic species inside the taxon *P. fuscus*. The Taxonomic Committee of the Societas Europaea Herpetologica recognized this in their latest assessment and elevated the taxa *balcanicus* and *vespertinus* to the taxonomic rank of a species ([11], see also [12]). Taking this recent change into consideration, the range of *P. fuscus* spans from Denmark in the North to Austria in the South (with a more southern disjunct population in northern Italy) and the Netherlands in the West to the European parts of Russia in the East. Therefore, our study area is located at the south-west border of the *P. fuscus* distribution range and the population belongs to the nominate subspecies *P. f. fuscus*.

Most of the studies on the common spadefoot toads investigate animals at the breeding sites, which is where they can be observed most easily [13–15]. Only very few studies investigated spadefoot toads in their terrestrial habitat [e.g. 16], despite the obvious importance of this habitat type for such a terrestrial toad [17]. In our study we used a novel approach; we investigated barn owl pellets for remains of *P. fuscus*, in order to analyze possible activity patterns of these animals in response to environmental parameters and during the year. It is known that common spadefoot toad individuals are found in barn owl pellets [18], however, systematic analyses focusing on common spadefoot toads are lacking. This is surprising as barn owl pellets have been used for decades as a crude but highly efficient monitoring method of small mammal communities (i.e. mice and shrews) [19]. In fact, the pellets from the presented study were also examined for small mammals, which allowed us to analyze small mammal communities and was published in a separate study [20].

In the current long-term study, we exemplify that barn owl pellet analysis can be used as an efficient method to indicate activity patterns of the common spadefoot toad.

## Methods

### Study area

**Fig. 1:**
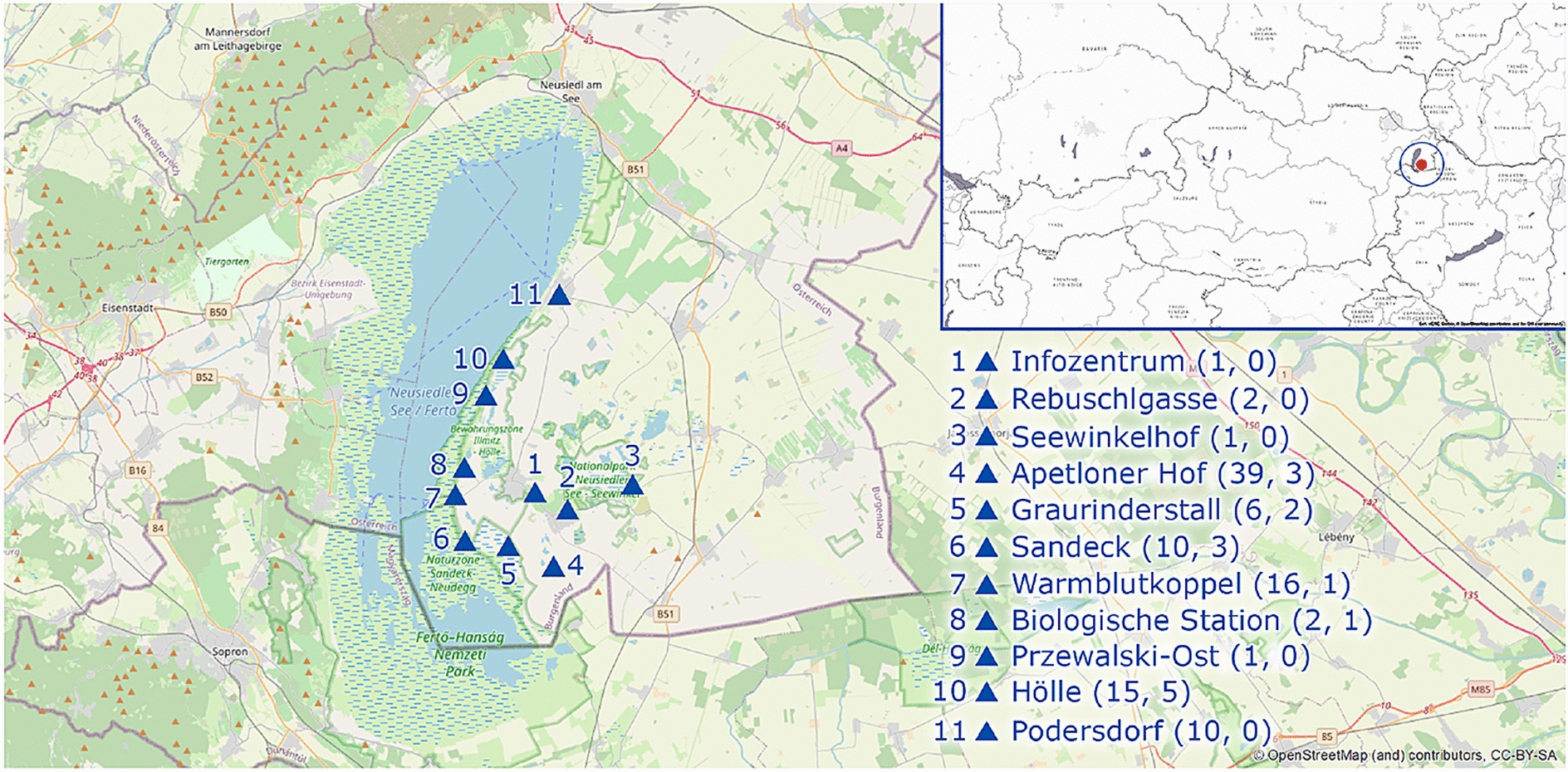
Sample locations in the study area at the East of the Neusiedler See (in the East of Austria, at the Hungarian border). Blue triangles show the sampling locations. At the right lower corner, the associated names and sample numbers (in parenthesis) are shown (first number in parenthesis: total number of samples, second number: number of samples with *P. fuscus* present)

The study area is located in the East of Austria very close to the Hungarian border, at the East of the Neusiedler See (Fig. 1). This area used to be dominated by a characteristic steppe environment with meadows, however, vineyards and other agricultural land are common at the present time. The area offers rich breeding habitats for amphibians ranging from periodic salt lakes to permanent freshwater ponds and lakes. The region was acknowledged as a world heritage site by the UNESCO in 2001 [21].

### Sample collection and species determination

We analyzed barn owl pellets from 2004 to 2016 for *P. fuscus* remains. Spadefoot toads are identified by their characteristic tailbones and frontal bones, which makes them easily distinguishable from other vertebrates [22]. Barn owls regurgitate such undigested remains in pellets, which were collected in the study area. The pellets were collected by a ranger (Vinzenz Waba) of the National Park Neusiedler See-Seewinkel who knew the barn owl roost sites. For each collection, all the pellets found at one roost site were combined. One collection trip to one site was therefore regarded as one independent sample. All remains are stored in the Natural History Museum Vienna in the Mammal Collection.

### Statistical analysis

In addition to the data on the number of individuals in the pellets we obtained environmental data from ZAMG (Zentralanstalt Meteorologie und Geodynamik), the national meteorological and geophysical service of Austria (measurements taken from the nearest measuring station: 47° 46′ 21′′ N, 17° 2′ 0′′ E, 118 m above the sea level of Adriatic Sea, ~ 8 km from study area). We performed all analyses in R [23], the script and raw data to reproduce the analysis, table and plots are available in the supplementary data (Additional file 2: Data and R scripts). In order to mathematically describe seasonal (cyclic) patterns throughout the year, we calculated the sine and cosine of the month (in radians, formula used: $\frac{2\pi }{12}\times month$ (for trigonometric functions see Pewsey et al. [24]). For bi-annual cycles we used the same approach calculating sine and cosine from the doubled radians for each month (later referred to as sine2 and cosine2). To analyze our data, we used a generalized linear model (GLM) using the function *glmmTMB* from the R package with the same name [25]. We used a Poisson distribution with zero inflation to model our data. We followed an automated model selection paradigm using the function *buildglmmTMB* (backward model selection based on likelihood ratio test) from the R package *buildmer* [26] to avoid overfitting and find the factors contributing to spadefoot toad abundance in the samples (which we assumed to be related to spadefoot toad activity). Our full model included: site, year, sum of precipitation per month, maximum precipitation in 24 h (in a month), snow (per month), minimum temperature (per month), mean temperature (per month), sine, cosine, sine2 and cosine 2 as well as all interaction terms. Tables were prepared using the *tab_model* function in the R package sjPlot [27]. We calculated predictions with 95 % confidence intervals using the function *ggpredict* from the R package *ggeffects* [28]. Plots were prepared using the *ggplot* function in *ggplot2* [29]. For all plots, we converted the trigonometric terms back into months and averaged them, in order to show the relevant effects on a more intuitive scale (e.g. months instead of cosine).

## Results and discussion

The final model, after model selection, included: maximum precipitation in 24 h, cosine, sine2, cosine2 and the interaction between cosine2 and precipitation (Table 1). Neither site nor year was included in the best model; this suggests that precipitation and the time of the year explained the observed variation in spadefoot toad occurrence better than the sampling year or location. This also means that we have no evidence for location specific *P. fuscus* hot spots nor a decrease or increase in abundance over the years. However, we want to highlight that all of this is based on relatively few toad specimens in the pellet samples (50 total individuals in 103 independent samples). We only observed toads in 4 out of 13 years, possibly suggesting that spadefoot toad activity can fluctuate dramatically between years, presumably based on favorable weather conditions. Hence, direct observation and long-term capture-recapture studies would be needed to gain insights in population dynamics and detect increases or decreases, as it has been successfully done in other studies [e.g. 30].

**Table 1:** Results for count model.

Predictors	*Pelobates fuscus*			
	Estimate	SE	z	*p*
Max. Prec. in 24 h	0.26	0.05	4.99	< 0.001
Cosine (month)	2.92	0.85	3.45	0.001
Sine2 (month)	− 3.97	1.19	− 3.32	0.001
Cosine2 (month)	− 5.16	1.55	− 3.32	0.001
Mean T (month)	0.23	0.11	2.13	0.033
Max. Prec. * Cosine2	0.14	0.05	2.91	0.004
Observations	103			

Shown are the estimate, standard error (SE), test statistics (*z*) and *p*-values (*p*)

**Fig. 2:**
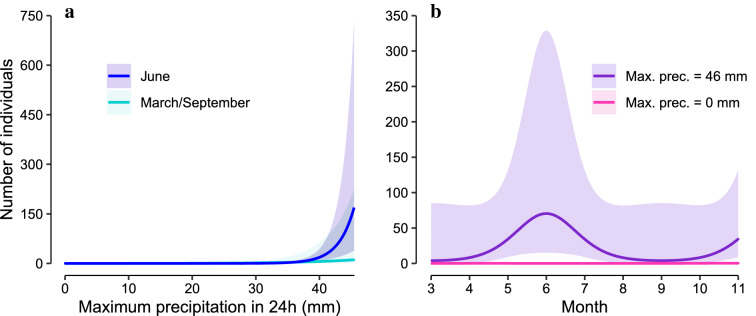
Model predictions for rain and month effects. Lines indicate the predictions, shaded area the 95 % confidence interval (CI), ranges of independent variable shown only include values with actual observations. Rain (i.e. maximum precipitation in 24 h) had significant main effects on spadefoot toad numbers, however, the effect was strongest in June (**a**). To visualize the seasonal effects, we back-transformed the trigonometric functions to months and averaged the effects, as well as the CI. We observed a striking increase of toad activity in June with intense rainfalls (**b**). Main effects of month and temperature only had minor effects (< 1 individuals) on toad activity (see Additional file 1: Fig S1)

Our analysis shows very clearly that spadefoot toads were more active (more often found in pellets) around heavy rainfalls (Fig. 2a; Table 1). It is important to note that the rainfall during a single day (24 h period) seems more important than the rainfall summed over a month, as the latter was not included in the final model. The dependence on rainfall is known from the literature, however, in our data this dependency seems to be especially striking during summer, while their main breeding season takes place from April to May [31]. Interestingly, it has been observed that spadefoot toads occasionally have a second breeding period in the summer months, in the event of major rainfalls [32]. We assume this is the reason for the interaction between rainfalls and cosine2. In other words, rainfalls in summer are predicted to lead to high spadefoot toad activity, which is likely indicating breeding activity (Fig. 2b). The main effects of the month show a slightly increased activity in Spring (the typical main breeding season [32, 33]) and Fall; in contrast, if rainfall remains at average levels they predict minimal toad activity in Summer (Additional file 1: Figure S1A). There is a small, but significant, trend of higher toad activity with increasing temperature, if all other factors stay at average levels (Additional file 1: Figure S1B). This is not surprising, as spadefoot toad breeding activity is known to be influenced by temperature [34]. It remains to be seen how climate change will impact spadefoot toad activity (and distribution). Increased temperatures might have minor positive effects, the expected drier conditions, however, will seriously challenge these populations and might lead to a loss of habitable areas for *P. fuscus* [35].

In conclusion, our analysis shows how pellet data can be used to identify activity patterns of spadefoot toads which have a rather hidden lifestyle. For the common spadefoot toads this method is very applicable, as the species are easily to identify from the remains and cannot be confused with other species and are often missed by other monitoring methods. The only prerequisite needed is an existing owl population. Luckily, the barn owl is a very common and widespread bird species around the world. Considering the effects seen in our study we expect other pellet studies with around 100 samples to be feasible. However, we would encourage newly initiated monitoring efforts to obtain samples regularly around the year, e.g. bi-monthly. Such regular sampling could increase the resolution of predictions and provide a more detailed account of spadefoot toad activity. The caveat of using pellets is that spadefoot toads only occur at low abundances in such samples, and if no toads are found, no analysis can be performed. Even if spadefoot toads are found in the samples, they will be few. It appears that only peak toad movement activities are represented in such data. In the present study the number of samples was quite different between years (min = 3, max = 18), which is certainly not what we would recommend for future studies. However, linear models including zero-inflation can overcome such inconsistencies as well as low numbers of detections, which are common for data derived from monitoring efforts. Given that pellet studies are still performed and analyzed nowadays and have been for a long time, a lot of pellet samples and data exist. Therefore, including determination of the spadefoot toad, could allow us to easily compare different populations around Europe. Thereby, presence/absence and activity patterns of this rare toad could be compared across different habitat types and pelobatid species.

## Supplementary Information

**Additional file 1: Figure S1. **There was still a remaining main month effect (lower activity in the hottest months) after precipitation effects were accounted for, however the effects were very weak (**A**). Toad abundance in the pellets slightly increased with mean temperature, also here effects were predicted to be very weak (**B**).

**Additional file 2:** Data and R scripts. The original data used and the R script to reproduce our analysis.

## Data Availability

All data generated or analyzed during this study are included in this published article and its additional file.
