# Supplementary figures and images for: Long‐term monitoring of common spadefoot toad activity in a European steppe using barn owl pellets
Source: J Biol Res (Thessalon). 2021 Feb 12;28:4. doi: 10.1186/s40709-021-00133-w (PMC7879639; doi:10.1186/s40709-021-00133-w)

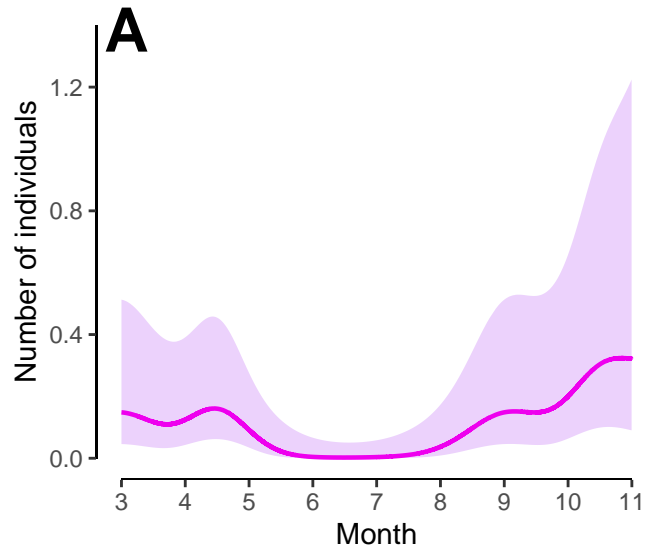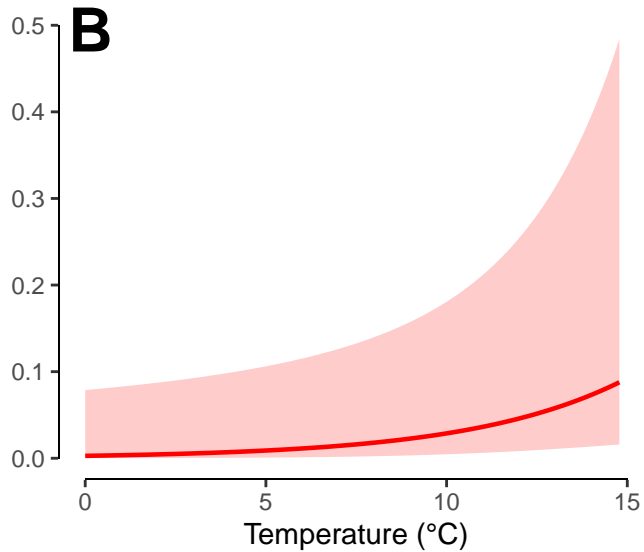

Supplement: Supplementary file 1 — Additional file 1: Figure S1. There was still a remaining main month effect (lower activity in the hottest months) after precipitation effects were accounted for, however the effects were very weak (A). Toad abundance in the pellets slightly increased with mean temperature, also here effects were predicted to be very weak (B). [file 40709_2021_133_MOESM1_ESM.pdf]
